# May-Thurner Syndrome in an Elderly Man

**DOI:** 10.7759/cureus.21611

**Published:** 2022-01-25

**Authors:** Sittinun Thangjui, Angkawipa Trongtorsak, Jerel M Zoltick, Adam Doyle

**Affiliations:** 1 Internal Medicine, Bassett Healthcare Network, Cooperstown, USA; 2 Internal Medicine, AMITA Health Saint Francis Hospital, Evanston, USA; 3 Cardiology, Bassett Healthcare Network, Cooperstown, USA; 4 Vascular Surgery, University of Rochester Medical Center, Rochester, USA

**Keywords:** iliocaval venous compression syndrome, cockett’s syndrome, iliac vein stent, pulmonary embolism, deep vein thrombosis (dvt), may-thurner's syndrome

## Abstract

May-Thurner syndrome (MTS) is a rare cause of deep vein thrombosis (DVT). This diagnosis is seldomly included in the differential diagnosis. The disease is defined as extraluminal iliac vein compression by the arterial system against bony structures in the iliocaval area. This occurs more commonly on the left side due to the unfortunate position of the proximal left iliac vein that runs between the right common iliac artery and spine. MTS is commonly presented in younger female patients with left unilateral proximal DVT. However, MTS is rarely reported in elderly patients. We present a case of a 69-year-old man with a diagnosis of MTS and further management with a venous stent.

## Introduction

Venous thromboembolism (VTE) incidence rate is approximately two per 1000 people in the United States with a mortality rate of up to 10% [1]. VTE can be categorized into provoked and unprovoked. This helps guide the treatment duration of anticoagulation, which is shorter in the provoked group. The unprovoked VTE group may require further testing for hypercoagulable disorders or occult malignancy depending on the risk factors. Apart from that, the anatomical deformity should also be considered in the differential diagnosis with May-Thurner syndrome (MTS) as a rare cause of VTE. This report presented a case of an elderly man with left leg swelling who was concerned for MTS.

## Case presentation

A 69-year-old Caucasian man presented with dyspnea on exertion and left leg swelling. He lived an active lifestyle and exercised regularly using a treadmill. He noticed swelling of his left legs for the past six days and experienced dyspnea on exertion during his exercise session, which led him to visit the emergency department. The patient had a past medical history of essential hypertension, hyperlipidemia, and benign prostate hyperplasia. His medication included losartan, hydrochlorothiazide, and atorvastatin. He never smoked and never used recreational drugs. On examination, his vital signs were normal, and oxygen saturation was 96% on ambient air. The cardiopulmonary exam was benign. Left lower extremity was warm with non-pitting edema from the proximal thigh to the ankle without tenderness and erythema. Dorsalis pedis and posterior tibial pulses were full bilaterally.

Initial laboratory tests included complete blood count with differential, basic metabolic panel, and troponin, and B-type natriuretic peptides were within normal limits. Electrocardiography showed normal sinus rhythm with a rate of 61 beats/minute without ST-T abnormality. Transthoracic echocardiography showed preserved left ventricular ejection function with mild left ventricular concentric hypertrophy without right ventricular systolic dysfunction. Computed tomography (CT) angiography of the chest with pulmonary embolism (PE) protocol showed bilateral segmental and subsegmental pulmonary embolism. Lower extremity venous duplex ultrasound was positive for acute occlusive deep vein thrombosis (DVT) left proximal and mid popliteal vein as well as proximal and mid peroneal vein. Hypercoagulable profile was negative for prothrombin gene mutation, factor V Leiden mutation, and cardiolipin antibody. The patient was treated with enoxaparin 1 mg/kg subcutaneously every 12 hours for two days and was able to discharge after three days of hospitalization. At discharge, apixaban 10 mg orally twice daily was prescribed for seven days, then 5 mg twice daily for lifelong due to unprovoked etiology. Cancer screening with colonoscopy and CT scan of the abdomen with contrast were negative for suspicious mass.

**Figure 1:**
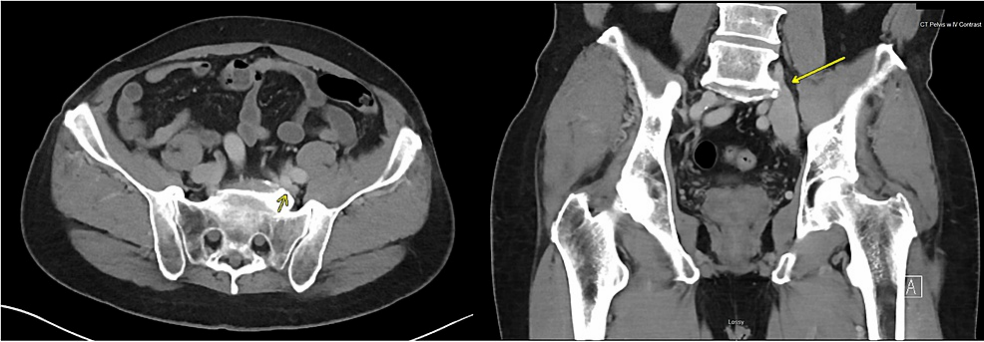
Left common iliac vein compression CT scan of pelvis with contrast showing extrinsic compression of the left common iliac vein between the left internal iliac artery and fifth lumbar spine (yellow arrows)

**Figure 2:**
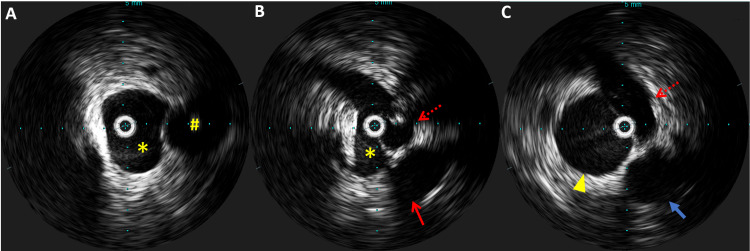
Intravascular ultrasound of left common iliac vein Intravascular ultrasound of iliac vein before venous stent placement. (A) Left common iliac vein (asterisk) and left common iliac artery (hashtag) at the proximal level to the compression; (B) left common iliac vein (asterisk) compressed by the left internal iliac artery (dotted arrow) at the level of external compression; (C) left external iliac vein (arrowhead) at the level of post obstruction and the thin head arrow showing left external iliac artery.

The patient was able to return to his baseline status and exercise regularly. His shortness of breath symptoms resolved. However, his left leg swelling had only minimal improvement. CT scan of pelvis with contrast was obtained due to a concern of anatomical obstruction of the left iliac vein. It showed a significant extrinsic compression upon the left common iliac vein between the left internal iliac artery and lumbosacral spine without pelvic vein thrombosis (Figure 1). The diagnosis of MTS was suspected. The patient underwent a left iliac vein angiography with a venous stent. A Wallstent (Boston Scientific, Massachusetts, United States) that is 22 mm in diameter and 70 mm in length was placed under intravascular ultrasound (IVUS) (Figure 2). The patient tolerated the procedure well. No acute complication was noted, and the left leg swelling was improved. Apixaban 5 mg orally twice daily was continued, and clopidogrel 75 mg orally once daily was started. Left leg swelling resolved, and the patient was able to return to regular exercise.

## Discussion

MTS, also known as iliocaval venous compression syndrome (IVCS) or Cockett’s syndrome, is defined as extraluminal iliac vein compression by the arterial system against bony structures in the iliocaval area [2]. The disease was associated with an eight-fold increase in the risk of left pelvic vein thrombosis and was originally described by May and Thurner in 1957 [3]. From 430 cadavers examined, 19% of adults had spur formation in the left common iliac vein at the junction with the inferior vena cava, but none of the embryo and newborn groups had the spur. The spur is an intraluminal callus-like formation, not part of the vein intima, consisting of loose connective fibrous tissue fed by protruding capillaries from the vasa vasorum of the vein, which forms after birth and is not part of the congenital anomalies. The spur formation is caused by chronic irritation of the vein due to compression of the proximal iliac vein by the iliac artery and the L5 vertebral body.

MTS commonly presents with unilateral left leg swelling or DVT in women in their 30s, particularly in the postpartum period. MTS’s risk factors include scoliosis, hypercoagulable disorders, contraceptive use, and prolonged dehydration. However, unilateral leg swelling and DVT, especially proximal lower extremity, caused by MTS in elderly patients was rarely diagnosed. A retrospective study of elderly patients with hip fractures, mean age of 81 years, showed that 34% of patients who developed DVT had baseline IVCS [4]. This resulted in an increased risk of DVT of up to 1.5- to two-fold. In general, iliac vein stenosis of at least 50% can be found in up to 10% of asymptomatic patients with a mean age of 55 years [5].

In recent years, the number of studies that reported MTS in elderly patients who are 60 years old and above has increased. Awareness and knowledge of IVCS play a vital role in detecting MTS in elderly patients. This is important because MTS requires additional treatment to reduce the recurrent rate of DVT and improve the quality of life [6]. A gold standard test for MTS is conventional venography with intravascular ultrasound [2]. An additional useful test is CT venography. This test provides data of the surrounding structure and helps identify the cause of obstruction better than the gold standard test [7]. Treatment for MTS includes treating the acute DVT with catheter-directed thrombolysis to prevent post-thrombotic syndrome [7]. Angioplasty with an endovascular stent, preferably a self-expanding stent, is recommended as a definite treatment after DVT resolution. Venous Wallstent (Boston Scientific) is a common stent used to treat MTS in the United States. It was approved by FDA in March 2020 with one-year stent patency of 86.6% [8]. A dedicated venous stent, the BARD® Venovo™, was introduced in 2016 with an 88.3% one-year stent patency [9]. To date, there is no consensus on the use of post-procedural antithrombotic therapy since there is no data from clinical trials to support the use of dual antiplatelet over single antiplatelet. The discussion about the risk and benefit of antiplatelet and anticoagulation use is important between the physicians and the patients.

## Conclusions

MTS or IVCS is an uncommon condition in elderly patients. However, physicians should be aware of MTS especially in patients with recurrent proximal left leg DVT. High suspicion for MTS is needed to pursue a proper workup for MTS using CT venography. After treating acute DVT, an endovascular venous stent is recommended as a preferred definite treatment.
